# Dataset on the synthesis and characterization of boron fenbufen and its F-18 labeled homolog

**DOI:** 10.1016/j.dib.2017.08.048

**Published:** 2017-09-05

**Authors:** Chun-Nan Yeh, Chi-Wei Chang, Yi-Hsiu Chung, Shi-Wei Tien, Yong-Ren Chen, Tsung-Wen Chen, Ying-Cheng Huang, Hsin-Ell Wang, You-Cheng Chou, Ming-Huang Chen, Kun-Chun Chiang, Wen-Sheng Huang, Chung-Shan Yu

**Affiliations:** a Department of Surgery, Liver Research Center, Chang-Gung Memorial Hospital at Linkou, Chang Gung University, Taiwan; b Department of Nuclear Medicine, Veterans General Hospital at Taipei, Taiwan; c Center for Advanced Molecular Imaging and Translation, Chang Gung Memorial Hospital, Taiwan; d Department of Biomedical Engineering and Environmental Sciences, National Tsinghua University, Hsinchu 300, Taiwan; e Department of Neurosurgery, Chang-Gung Memorial Hospital at Linkou, Chang Gung University, Taiwan; f Department of Biomedical Imaging and Radiological Sciences, National Yang-Ming University, Taipei, Taiwan; g Department of Medical Radiology, Yang-Ming University at Taipei, Taiwan; h Department of Surgery, Chang-Gung Memorial Hospital at Keelung, Chang Gung University, Taiwan; i Institute of Nuclear Engineering and Science, National Tsing-Hua University, Hsinchu 300, Taiwan

**Keywords:** Setup, NMR spectra, Coupling constant, Binding, Survival

## Abstract

The data presented in this article are related to the research article entitled “Synthesis and Characterization of Boron Fenbufen and its F-18 Labeled Homolog for Boron Neutron Capture Therapy of COX-2 Overexpressed Cholangiocarcinoma”. The contents of the data article include 1) the set up for performing *in vitro* binding assay, 2) ^1^H-, ^13^C- and ^19^F-NMR of compounds described in main text, 3) HPLC chromatogram of the fluorination mixtures, 4) data of *in vitro* stability test, cell survival assay, western blot and PCR analysis, 5) the modules for fixing the two CCA rats for BNCT, and 6) bar diagram for tumor reduction using [^18^F]FDG-PET 24 h post treatment with BNCT.

**Specifications Table**

**Table:** 

**Subject area**	Chemistry, Biology
**More specific subject area**	Spectroscopy, Chromatography, Bioassay
**Type of data**	Table, Chromatogram (HPLC), Illustration, Graph, Figure
**How data was acquired**	Spectra of NMR of ^1^H, ^13^C and ^19^F were generated from NMR experiments. Survival assay was performed by MTT protocol. Statistical analysis of PET imaging was analyzed. Setup for binding assay and BNCT module was described. Data of mRNA and protein expression level were described.
**Data format**	Raw, Analyzed.
**Experimental factors**	Samples have been well prepared, purified and characterized.
**Experimental features**	The relevant data of cell survival assay, HPLC chromatogram and analytic data were determined.
**Data source location**	Hsinchu, Taoyuan and Taipei, Taiwan
**Data accessibility**	All the data is with this article.

**Value of the data**

• NMR spectra of the target compounds and intermediates are useful for structural characterization.
• Set up for binding assay is useful for other researchers to perform the relevant binding assay design.
• Expression level of protein and mRNA of COX-2 in HuCCT1 is useful for future selection of patients for BNCT study.
• Module for performing neutron irradiation is useful for ensuring the measurement correctness.

## 1 Data

The data set of this article provides information on structural data, separation data, and setup. Figs. 1 and 8 show the binding and BNCT irradiation setups, respectively. Fig. 2 shows the HPLC chromatogram. Fig. 4, Fig. 5, Fig. 6, Fig. 7 show the bioassay data. Fig. 9 shows the statistics of imaging data. Fig. 3 and the rest data show the NMR and mass data.

**Fig. 1:**
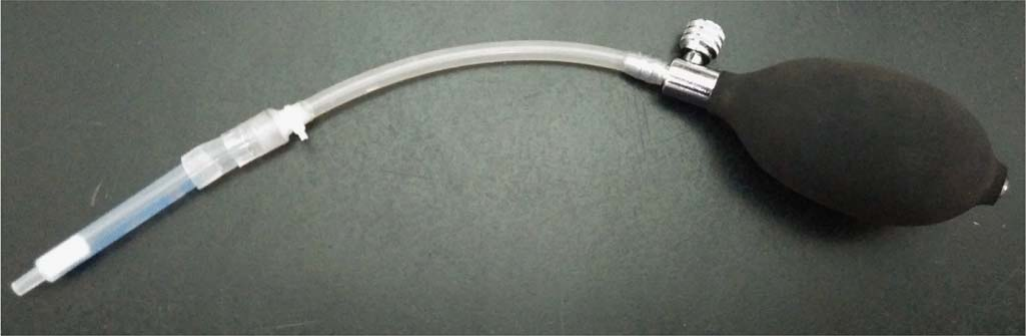
The setup used for performing binding experiment. The front part of this kit is purchased from disposable extraction column which is connected to the blood pressure ball using a silicon tube. Mixtures containing enzyme, ligand and tracer were firstly transferred to the extraction column. The pressure ball was then connected through the silicon tube. After the first portion of mixture was eluted, a second portion of EtOH was used to wash the residual mixture under the pressurizing. The two portions were combined as the mobile phase for counting the radioactivity. Radioactivity of the solid support and the mobile phase were individually counted and used to generate the binding curves.

**Fig. 2:**
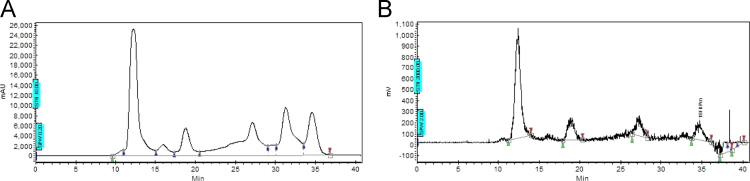
HPLC chromatogram comprising two sequential channels of UV- (A) and Radio-window (B) for the mixtures obtained from the radiofluorination of fenbufen boronopinacol **4**. Fractions corresponding to *ortho*-FFBPin ***o-*****6** at *t*_*R*_=26.5–28.6 min and *meta*-FFBPin ***m***-**6** at *t*_*R*_=33.5–35.5 min were isolated. Eluting conditions: isocratic mode, RP-18 column (Develosil ODS-7 5 μm 20×150mm), 85% CH_3_CN (aq.) for FFBPin **6** at a flow rate of 3 mL/min with λ=254 nm. Peak from 12 to 15 min might be the fluorodeboronated byproducts which have not been discussed in this paper.

**Fig. 3:**
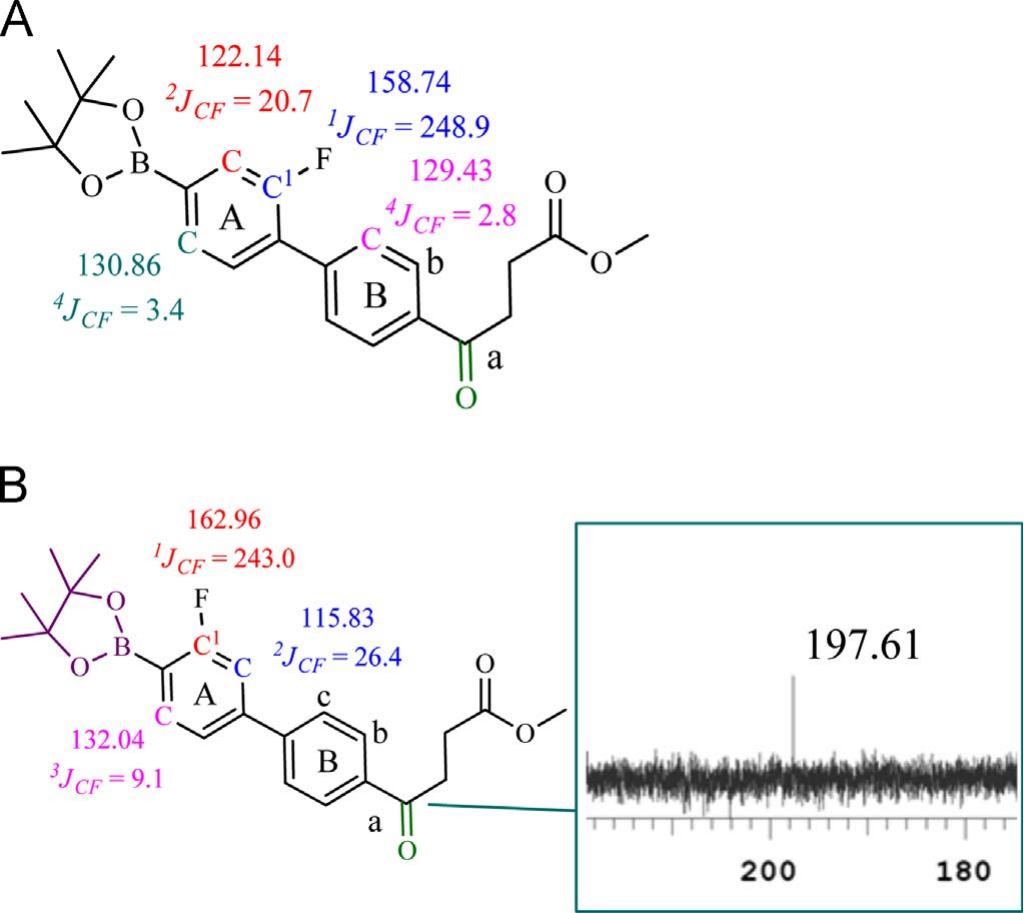
^13^C-^19^F coupling data of (A) *m*-FFBPin, ***m***-**6** and (B) *o*-FFBPin, ***o***-**6**. Coupling constants derived from different ranges of carbon and fluorine generated the typical constants that agreed with the literature as described in the main text. Fluorination was not expected to occur on the ring B because of the electron withdrawing effect by the carbonyl group.

**Fig. 4:**
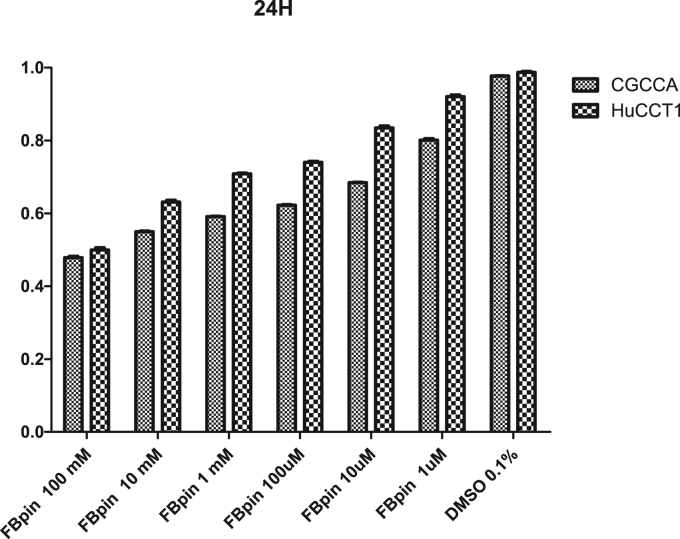
Cell survival assay after treatment of FBPin **4** for 24 h. The cell toxicity was assessed by a viability assay, MTT (3-(4,5-dimethyl-thiazol-2-yl)-2,5-diphenyl tetrazolium bromide) reduction. In brief, T98 (human glioma cell line) cells were plated in the 96-well plate (5000/well) and maintained at 37 °C, 5% CO_2_ incubator with MEM, 10% fetal calf serum (Gibco, USA). After 24-h or 48-h pulse with FBPin, 50 μL of MTT (5 mg/mL) was added to each well for four hours. After removal of medium, 100 μL of DMSO was added to each well and the optical absorbance was determined by a spectrophotometry using a plate reader at 570 nm.

**Fig. 5:**
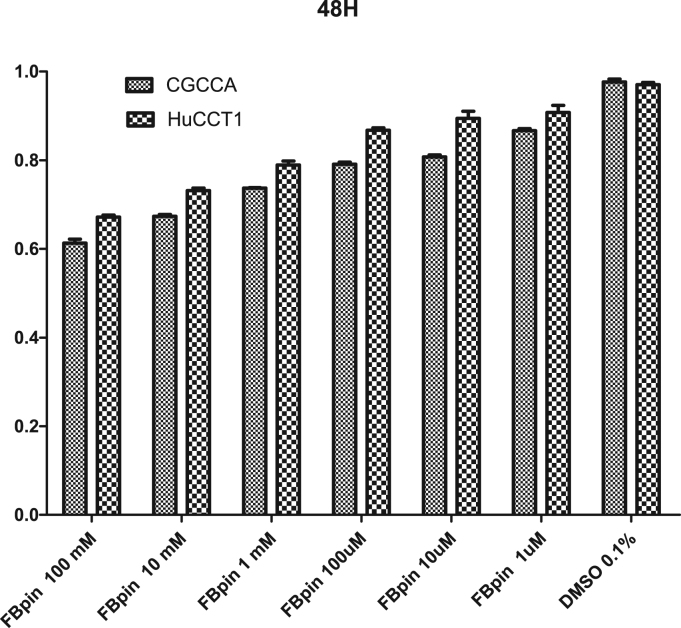
Cell survival assay after treatment of FBPin **4** for 48 h. The same methods as that described in Fig. 4 were used.

**Fig. 6:**
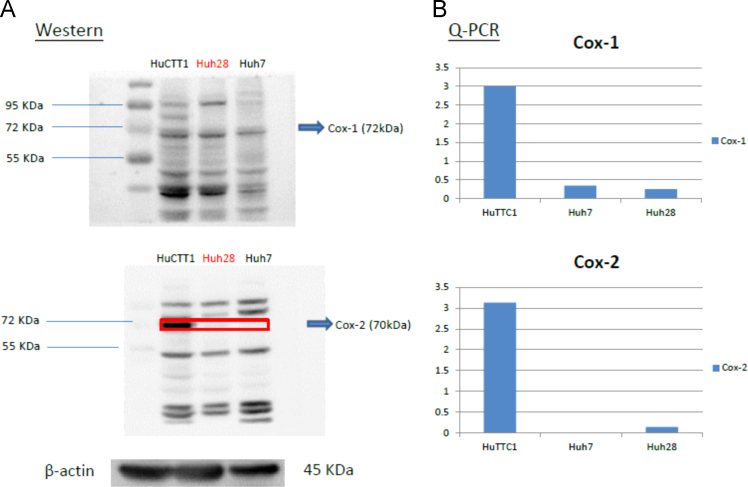
Expression level of protein and mRNA in terms of Western blot and PCR analysis, respectively, for the three cell lines.

**Fig. 7:**
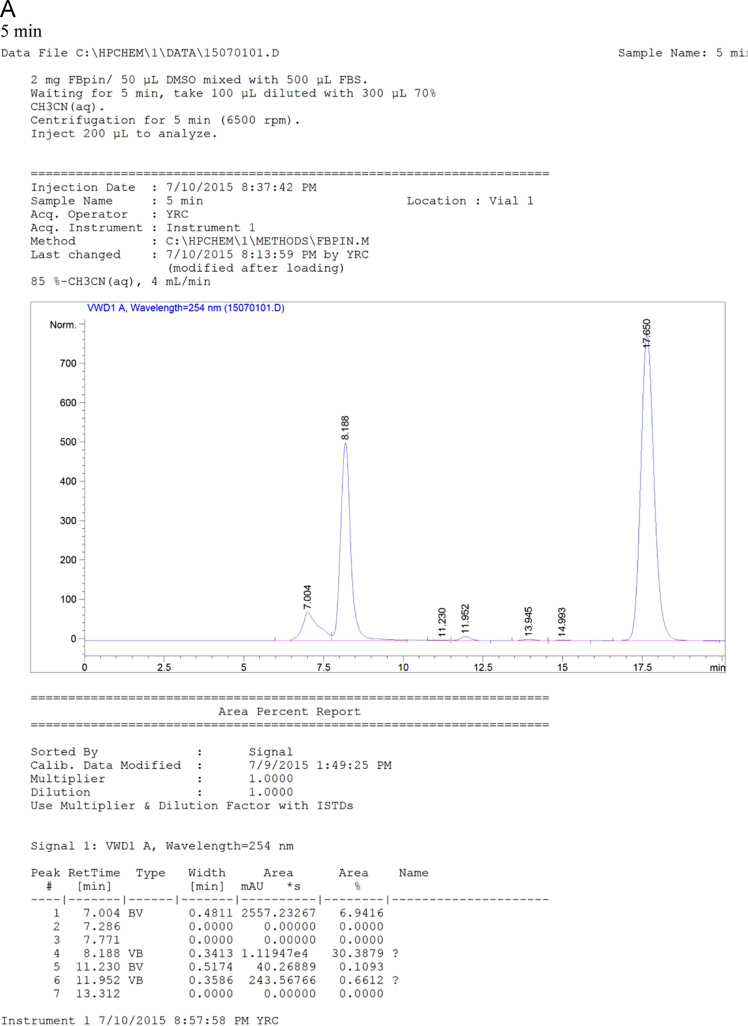

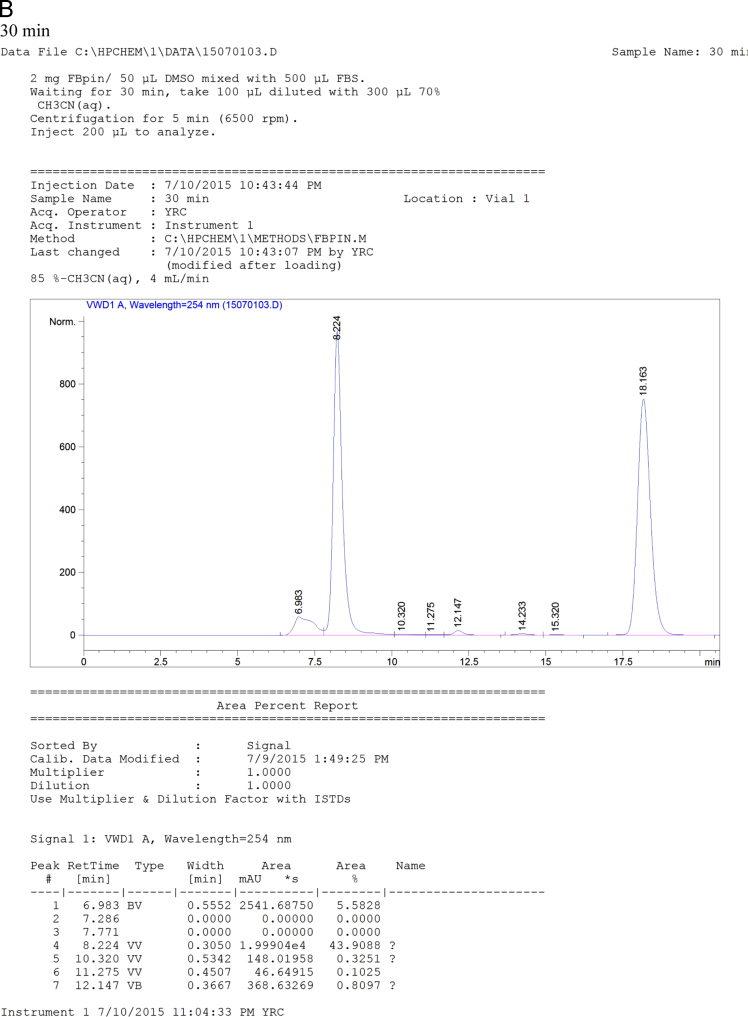

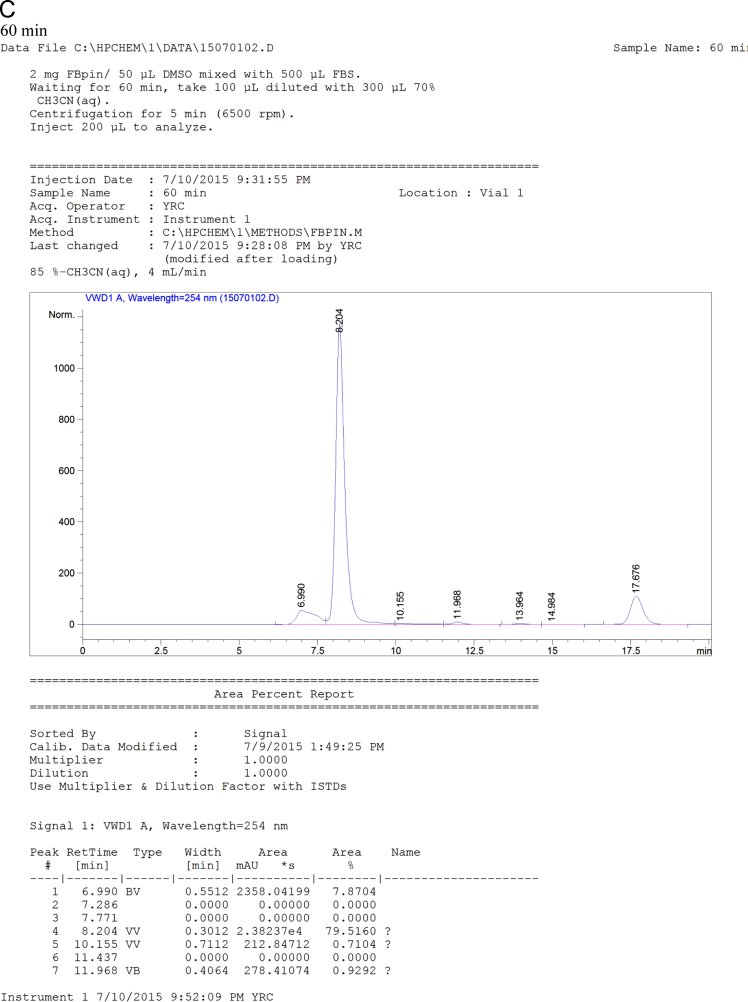

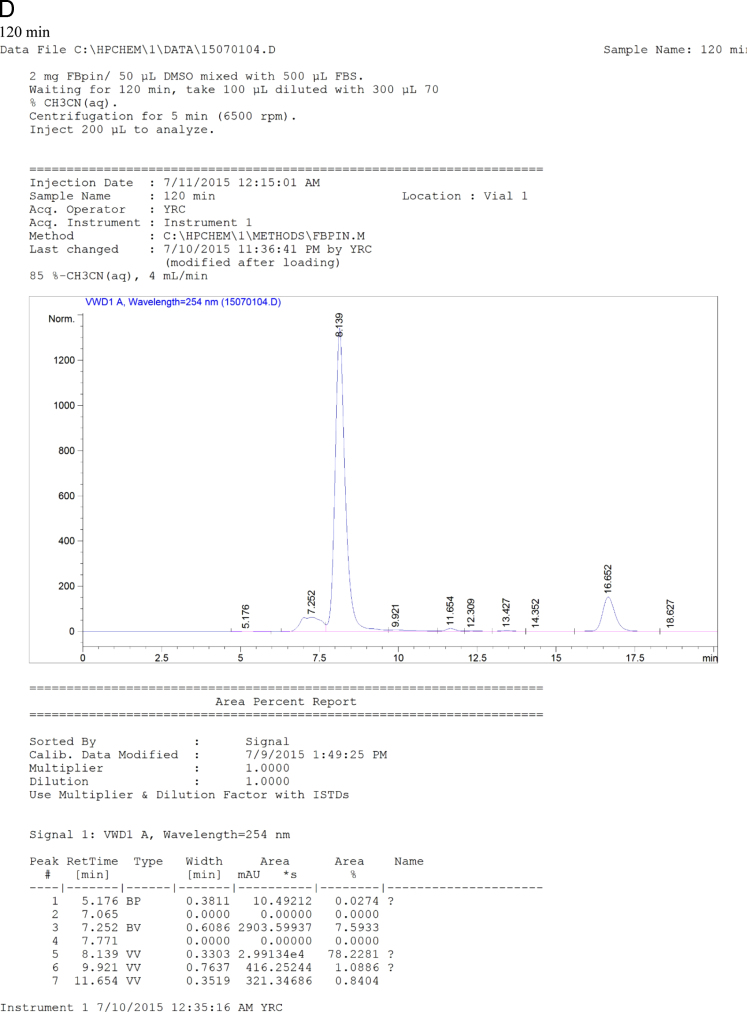
HPLC chromatogram of the mixtures of FBPin **4** and FBS at various time points. The HPLC system comprised Agilent 1100 series quarternary pump, injection loop (0.5 mL) and UV detector at 260 nm, variable wavelength detector (G1314A) and a preparation column : Develosil ODS-7 5 μm 20×150mm.

**Fig. 8:**
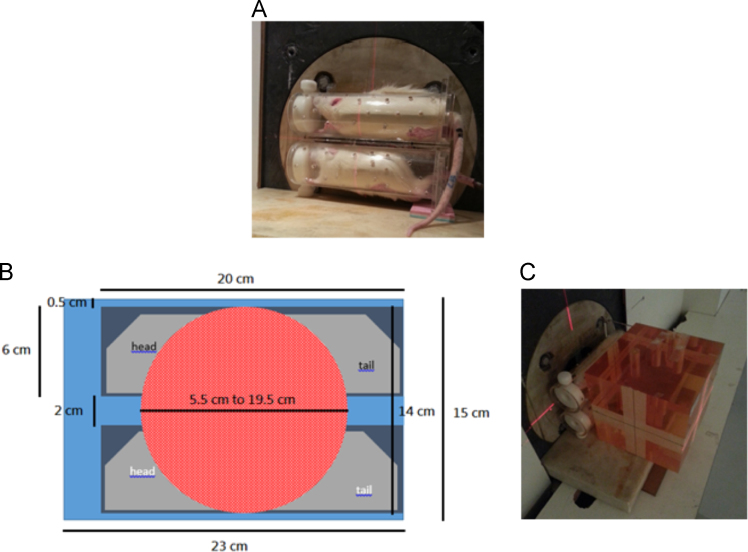
(A) and (B) The set up used to fix the two CCA rats for performing simultaneous BNCT and NCT was covered by the neutron beam line. (C) The plastic modules configured such that the neutron beam line could be more focused on the targeting zone.

**Fig. 9:**
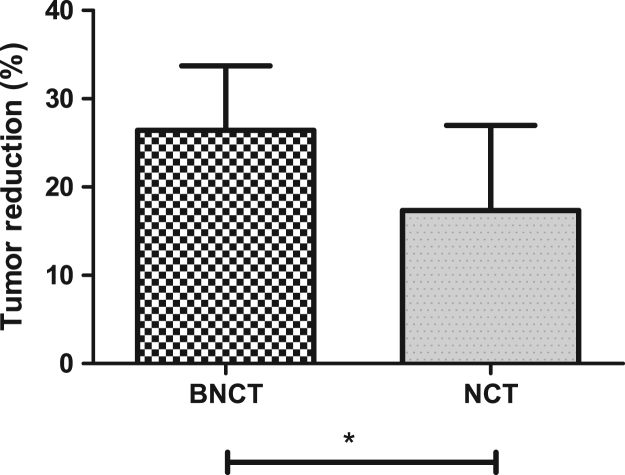
Comparison of the reduction of uptake of [^18^F]FDG-PET 24 h post treatment with BNCT (4 tumor sites from each of two CCA rats) and NCT (3 tumor sites from each of another two CCA rats. BNCT: 26.42±2.58 (*n*=8), NCT: 17.37±3.92 (*n*=6), P=0.0336. PET scanning experiments were performed after each treatment. The ROIs’ changes after treatments were expressed in bar diagrams.

## 2 Experimental design, materials and methods

Chemical synthesis and radiochemical synthesis provided the target compounds. Chemical structures were characterized using ^1^H-, ^13^C- and ^19^F-NMR and low-resolution and high-resolution mass spectrometry (LR-MS, HR-MS). Meta- and ortho-fluoro fenbufen compounds are differentiated based on ^13^C-^19^F coupling constants. Radiofluorination gave the meta- and ortho-FFBPin, ***m***-**6** and *o*-FFBPin, ***o***-**6**. The two isomers were obtained in limited amounts of less than 3 mg. ^1^H-NMR were not easily used to identify the J_F,H_ of aromatic couplings because they are similar to the values of J_H,H_. The ^13^C-^19^F coupling constants from different bond ranges could be used to identify the fluoro position. Binding assay using the set up as shown in Fig. 1 was performed in a compact manner through a team work. One member is responsible for mixing the enzyme, tracer and ligand and for transferring the mixture. The other member pumped the mixture through the silica cartridge. The third member was responsible for controlling the timing. The rest work regarding the counting of the activity was done by the fourth member. The binding experiments were performed for COX-1 and COX-2, sequentially. The laborious pumping of the mixture for more than 30 samples was carried out by two members. The statistic error of the binding data was mainly due to this stage. Three HPLC systems were employed for the study. The HPLC system that was performed for purification of the cold fluorinated compounds, binding assay and stability assay comprised Agilent 1100 series quarternary pump, injection loop (0.5 mL) and UV detector at 260 nm, variable wavelength detector (G1314A) and a preparation column : Develosil ODS-7 5 μm 20×150mm. The study was carried out in the Medicinal Chemistry Laboratory of National Tsinghua University (NTHU). Purification and analysis of radiofluoro labeled compounds were performed using HPLC system in Taipei Veterans General Hospital. The two HPLC setups for both purification and quality control of the radiofluoro labeled compounds have been described in the main text. Samples that were ionized as cationic species by complexation with H^+^, Na^+^, and K^+^ through electrospray ionization were analyzed with Low Resolution (LRMS) and High Resolution Mass Spectrometry (HRMS). Isotopic patterns of the compounds were used to assist the identification of expected molecular mass.

