# Supplementary material for: Dataset on the synthesis and characterization of boron fenbufen and its F-18 labeled homolog
Source: Data Brief. 2017 Sep 5;15:174–202. doi: 10.1016/j.dib.2017.08.048 (PMC5633353; doi:10.1016/j.dib.2017.08.048)
Supplement: Supplementary file 1 — Supplementary material [file mmc1.docx]

Chung-Shan Yu

Associate professor, PhD

Tel: 886-3-5751922; Fax: 886-3-5718649; E-mail: csyu@mx.nthu.edu.tw

July 24^th^, 2017

I, Chung-Shan Yu, represent all the coauthors to declare that there are no conflict of interest regarding the paper: Synthesis and Characterization of Boron Fenbufen and its F-18 Labeled Homolog for [Boron Neutron Capture Therapy](https://www.google.com.eg/url?sa=t&rct=j&q=&esrc=s&source=web&cd=2&cad=rja&uact=8&ved=0ahUKEwie4vn2pKrNAhWLuhoKHaAYAjAQFggkMAE&url=http%3A%2F%2Fwww.ncbi.nlm.nih.gov%2Fpubmed%2F8951358&usg=AFQjCNGjY7vXfMm2HNHpa4uaQlVaaUSjCw&sig2=7mZ5wF0Z35HsfsyWBdYfUA&bvm=bv.124272578,d.d2s) of COX-2 Overexpressed Cholangiocarcinoma.

Sincerely,


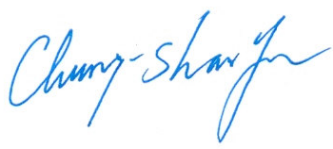


Chung-Shan Yu
